# 

**Published:** 1893-03-18

**Authors:** 


338, Vol. XIII.—March 18th, 1893.

[Registered at the General Post Office as a Newspaper for transmission in the United Kingdom and Abroad.]

Per Annum, 8s. 6d., post free.

Monthly Parts, 6d.; post free, 8d.

Ditto per Annum; 8s., post free.

